# Differences in muscle quality, muscle strength, and functional motor performance between people after stroke and age- and sex-matched apparently healthy adults: a cross-sectional study

**DOI:** 10.3389/fneur.2025.1641859

**Published:** 2025-09-09

**Authors:** Natalia Domínguez-Sanz, Paula Camelia Trandafir, Ana Beatriz Bays-Moneo, Ana María Insausti Serrano, Robinson Ramírez-Vélez

**Affiliations:** ^1^Departamento Ciencias de la Salud, Facultad Ciencias de la Salud, Universidad Pública de Navarra (UPNA), Tudela, Navarra, Spain; ^2^Department of Statistics, Computer Science and Mathematics, Universidad Pública de Navarra (UPNA), Pamplona, Spain; ^3^Institute of Advanced Materials and Mathematics2 (INAMAT2), Universidad Pública de Navarra (UPNA), Pamplona, Spain; ^4^Navarrabiomed, Hospital Universitario de Navarra (HUN), Universidad Pública de Navarra (UPNA), IdiSNA, Pamplona, Spain; ^5^CIBER of Frailty and Healthy Aging (CIBERFES), Instituto de Salud Carlos III, Madrid, Spain

**Keywords:** stroke, muscle thickness, muscle strength, echo intensity, ultrasound

## Abstract

**Introduction:**

After stroke, muscle quality deteriorates due to neuromuscular impairments, disuse, and ageing-related changes. While prior studies have examined global muscle alterations, less is known about regional differences in muscle quality across specific limb compartments, and how these differences relate to strength and functional performance. Understanding whether anterior and posterior compartments of the upper and lower limbs are differentially affected may help target rehabilitation strategies.

**Methods:**

This cross-sectional study investigated regional differences in muscle quality between people after stroke and age- and sex-matched apparently healthy adults, focusing on the anterior and posterior compartments of the upper and lower limbs. Muscle quality was assessed using ultrasound-derived muscle thickness (MT) and echo intensity (EI), alongside motor function (Modified Ashworth Scale), muscle strength (handgrip, knee flexion, and extension), and anthropometric measures. Analyses of covariance, adjusted for age, sex, body mass index, Mini Nutritional Assessment score, and Mini-Mental State Examination score, were conducted to compare the less affected and affected sides in people after stroke with apparently healthy controls. In total, 102 participants were assessed: people after stroke (*n* = 49) and apparently healthy controls (*n* = 53).

**Results:**

People after stroke exhibited significantly higher EI values in the biceps brachii (BB), rectus femoris (RF), tibialis anterior (TA), and gastrocnemius (GS) on both the affected and contralateral side (*p* < 0.05). MT values of the BB (*p* < 0.001), TA (*p* < 0.001), and GS (*p* < 0.001) on the affected side were significantly lower than those in controls. EI values of the BB and RF were negatively correlated with handgrip and knee extension strength, respectively, while MT of these muscles showed strong positive associations with strength outcomes (*p* < 0.001). Similar trends were observed for knee flexion.

**Discussion:**

Distinct regional patterns of muscle quality loss were observed in people after stroke, with lower MT and higher EI values correlating with reduced strength. These findings highlight the importance of assessing both structural and functional parameters across limb regions to better understand muscle deterioration and guide targeted interventions in ageing and post-stroke populations.

## 1 Introduction

Globally, stroke is the second leading cause of death, and the projection for 2,030 indicates that its incidence rate will increase (1). One quarter of stroke survivors suffer from severe motor disability at ninety days of onset (2). Beyond neurological deficits, stroke survivors show a markedly increased prevalence of sarcopenia, often with a disproportionate reduction in muscle mass on the affected side compared with the contralateral side (2, 3). This condition is characterized by severe abnormalities in muscle mass, such as muscle weakness and atrophy, particularly in the affected limbs (2). In fact, reductions in motor unit numbers in the paretic limb can occur within hours of stroke onset, with detectable losses reported as early as 4 h post-event, and these alterations often persist into the chronic phase (3).

Muscle strength has traditionally been the most widely used parameter for evaluating muscle status; however, the importance of muscle mass and quality is increasingly recognized (4–6). Chon et al. (7) reported that stroke-related sarcopenia involves not only muscle atrophy but also qualitative alterations, including increased intramuscular adipose tissue (IMAT) and changes in fiber composition, which differ between the paretic and contralateral sides. Akazawa et al. (8) further demonstrated that, in chronic non-ambulatory stroke survivors, reduced quadriceps muscle mass and elevated IMAT content are strongly associated with diminished muscle strength. More recently, Pradines et al. (9) showed that spastic myopathy—characterized by muscle stiffness, contracture, and altered architecture—significantly limits active movement and reduces ambulation speed in individuals with chronic spastic paresis.

This decline in muscle quality in both upper and lower extremities after stroke contributes to reduced performance in activities of daily living (ADL) and functional deficits (10–13). Ishimoto et al. (14) found that greater IMAT in the quadriceps over time is linked to declining gait independence among convalescent stroke patients, highlighting the negative impact of deteriorating muscle quality on mobility. Similarly, Thielman and Yourey (15) used ultrasound imaging to demonstrate that increased echogenicity in spastic upper-limb muscles correlates with impaired motor function. Nelson et al. (16) observed that chronic hemiparetic stroke patients display shortened fascicle lengths in both the biceps and triceps brachii on the paretic side, changes associated with motor impairment and functional limitations. Collectively, these findings indicate that post-stroke muscle alterations extend beyond atrophy, encompassing fat infiltration, architectural remodeling, and compositional changes that directly impair motor control and hinder functional recovery.

As mentioned above, muscle quality has received significant attention in recent years as an indicator for muscle assessment. Using computed tomography (CT), Ryan et al. (17) found that IMAT on the paretic side was ~25% higher than on the non-paretic side, and higher intermuscular fat levels have been linked to reduced strength (18, 19). Therefore, both muscle mass and quality should be considered when evaluating muscle strength. Muscle quality can be assessed by CT, MRI, or ultrasound (20). In this line, ultrasound-derived echo intensity (EI) has emerged as a widely used surrogate marker, reflecting changes in intramuscular composition—particularly increased fat and fibrosis—that are associated with impaired contractile efficiency (16). Although EI does not directly capture neural activation or fiber-type distribution, it is practical, reliable, and correlates strongly with functional outcomes in stroke and ageing populations (16). Beyond the well-documented loss of muscle mass and strength, increased IMAT is now recognized as a hallmark of muscle deconditioning in individuals after stroke (15–18, 20).

Muscle strength, mass, and quality are key indicators of overall muscle condition. Assessing changes in these parameters after stroke—particularly through ultrasound evaluation of multiple muscles in both apparently healthy adults and in the affected and contralateral limbs of people post-stroke—may provide valuable insights to enhance the effectiveness of rehabilitation strategies. However, much of the current literature is limited by small sample sizes or the use of advanced imaging modalities, such as MRI, that are costly and less feasible in routine clinical practice (21). Structural changes that impair muscle quality and functional motor performance can hinder motor recovery and reduce independence in people after stroke. In clinical practice, comprehensive characterization of muscle quantity and quality in both limbs is essential to guide personalized rehabilitation.

Therefore, the present study aimed to (i) investigate ultrasound-derived measures of muscle quality—muscle thickness (MT) and echo intensity (EI)—in four muscles (biceps brachii, rectus femoris, tibialis anterior, and the medial head of the gastrocnemius), and (ii) examine the associations between these parameters and limb-specific strength (handgrip and knee extension) after adjusting for age, sex, nutritional status, and cognitive function. We hypothesized that people after stroke would exhibit significantly impaired muscle quality—characterized by lower MT and higher EI—compared with age- and sex-matched apparently healthy adults, with alterations present in both upper- and lower-limb muscles, more pronounced on the affected side, and partially associated with reduced limb-specific strength after controlling for potential confounders.

## 2 Methods

### 2.1 Study design, participants, and ethics

This cross-sectional study recruited participants diagnosed with ischaemic or haemorrhagic stroke who were receiving rehabilitation treatment at the Asociación de Daño Cerebral de Navarra (ADACEN) between January 2024 and February 2025. People after stroke were recruited from local rehabilitation centres and stroke associations. Inclusion criteria were age ≥40 years, diagnosis of unilateral stroke, and ≥6 months since stroke onset. Non-inclusion criteria were substantial cognitive deficits, severe aphasia, cerebellar involvement, and any pre-existing conditions affecting walking function. Apparently healthy adults were recruited through community advertisements and local outreach initiatives. Inclusion criteria: age ≥40 years and independent ambulation. Non-inclusion criteria were the presence of orthopaedic, neurological, or chronic pain conditions, and a history of orthopaedic or soft-tissue surgery involving the upper arm, forearm, thigh, or lower leg (e.g., tendon repair, joint arthroplasty, compartment fasciotomy). The protocol was approved by the ethics committee of the Universidad Pública de Navarra (IRB No. PI-023/20). Written informed consent was obtained from all participants prior to data collection.

### 2.2 Muscle quality measurement

Ultrasound images were obtained using B-mode ultrasound imaging (Esaote MyLab™50; Esaote S.p.A., Genova, Italy) with a multi-frequency linear transducer (4–15 MHz). The following settings were applied for all measurements: frequency of 45 Hz, gain of 55 dB, and depth of 45–50 mm. Any optional post-processing within the software was disabled, and time gain compensation buttons were kept in their neutral positions. Transmission gel (Ultrasound GEL® Ref: 33273; Gima s.p.a Laboratories, Inc., Gessate, Italy) was used for all scans to enhance acoustic contact, and minimal pressure was applied to partially visualize the muscle border. Images were obtained for the BB, rectus femoris (RF), tibialis anterior (TA), and GS muscles on apparently healthy subjects, as well as on the less affected and affected sides of people after stroke. All ultrasound assessments were performed by the same sonographer, who had 4 years of experience and formal certification in advanced musculoskeletal ultrasound from “Centro ATM Fisioterapia, San Sebastián, Spain.” Examinations were conducted at the participants’ bedside with the individual in a standardized supine or sitting position whenever possible. Detailed protocols for patient posture and scan sites for each muscle are provided in Supplementary Table S1. Still images were captured in both sagittal and transverse planes, followed by panoramic images for comprehensive visualization. The selection of the four muscles in this study was intentional and based on both clinical and methodological considerations. The BB was chosen as a key representative of the anterior compartment of the upper limb, frequently affected by post-stroke weakness, altered motor control, and spasticity, particularly in elbow flexion (16). The RF represents the anterior compartment of the lower limb and is essential for knee extension and functional mobility, including walking and transfers (13, 15). The TA, in the anterior compartment of the lower leg, plays a critical role in ankle dorsiflexion, gait initiation, and foot clearance, functions often impaired after stroke (18, 19). The GS, part of the posterior compartment of the lower leg, is vital for plantarflexion and propulsion during gait, and is frequently affected by post-stroke spasticity (5). Together, these muscles cover anterior and posterior compartments of both upper and lower limbs, enabling a regional comparison that captures potential heterogeneity in muscle quality deterioration across the body. Furthermore, all four muscles are accessible for reliable ultrasound imaging, facilitating standardized measurement in clinical and research settings. Each site allows standardized probe placement and has demonstrated good reliability in post-stroke ultrasound studies (5).

Muscle architecture parameters were estimated in two ways. First, muscle quality was expressed using the index of EI (a.u.) values for each muscle, analysed with ImageJ, version 1.37 (National Institutes of Health, Bethesda, MD, USA). Regions of interest were set to include as much of the muscle as possible, excluding any visible fascia and/or bone. The EI of individual pixels was expressed on an 8-bit greyscale ranging from 0 (black) to 255 (white), and the mean EI value of each pixel within the region of interest was obtained. The mean EI within the region of interest was calculated for each image, and the average EI for each muscle was used in subsequent analyses. Higher scores indicated increased intramuscular fat and interstitial fibrous tissue, as proposed by Young et al. (22) We calculated the mean EI from limbs (EI limbs-1 and EI limbs-2) using the following equations:

$\mathrm{EI}\;\text{limbs}-1=[\begin{array}{l} (\mathrm{EI}\;\text{in}\;\mathrm{BB})+(\mathrm{EI}\;\text{in}\;\mathrm{RF}),(\mathrm{EI}\;\text{in}\;\mathrm{TA}),\\ \;\text{and}\;(\mathrm{EI}\;\text{in}\;\mathrm{GS}) \end{array}]/4$

EIlimbs−2=[(EIinRF),(EIinTA),and(EIinGS)]/3

The reliability of this methodology was established by Caresio et al. (23), and Yoshiko et al. (24), supporting the validity of our approach. Second, MT was measured as the minimum distance between the inferior border of the superficial aponeurosis and the superior border of the deep aponeurosis. MT values were recorded in cm. Each muscle was scanned three times, and the average of these measurements was calculated and used for further data analysis. All measurements were performed by the same investigator to minimize interobserver variation.

### 2.3 Upper and lower limb muscle strength measurements

Grip strength was measured according to the standardized position recommended by the American Society of Hand Therapists (25). The participants were seated with the shoulder in adduction and neutral rotation, the elbow flexed at 90°, the forearm in a neutral position, and the wrist positioned between 0° and 30° extension and 0° and 15° ulnar deviation. Isometric knee extension and knee flexion strength were measured using a handheld dynamometer (MicroFET 2; Hoggan Health Industries, West Jordan, UT, USA). The participants were instructed to produce a maximum isometric quadriceps contraction for 3 to 4 s. The same physical therapist conducted two measurements. Prior to testing, the procedure was demonstrated, and participants performed two rehearsals to familiarize themselves with the protocol. Measurements were expressed in kilograms. Additionally, a digital dynamometer (Takei Scientific Instruments Co., Tokyo, Japan) was used to measure grip strength. Ankle plantarflexion and dorsiflexion torque were not assessed because spastic equinus limited reliable range of motion and prevented secure limb fixation during testing.

### 2.4 Anthropometric, clinical, and motor function measurements

We also recorded age, sex, weight, height, body mass index (BMI), Barthel Index, MAS for assessing spasticity, Mini Nutritional Assessment (MNA) for screening and diagnosing malnutrition, Mini-Mental State Examination (MMSE) for cognitive assessment, and time since stroke. Health-related quality of life was measured using the Spanish version of the EuroQol-5D (EQ-5D) instrument. In addition, the EQ-5D includes a visual analogue scale (VAS), through which respondents report their perceived health status, rated from 0 (the worst possible health status) to 10 (the best possible health status). The ability to perform selected ADL was assessed using the Barthel Index. This instrument comprises 10 items (tasks), with total scores ranging from 0 (worst mobility in ADL) to 100 (full mobility in ADL), (26) and it demonstrates adequate clinimetric (clinical measurement quality) properties in stroke rehabilitation (27). The Modified Ashworth Scale (MAS) is a performance-based scale used to assess level of impairment and everyday motor function in patients with stroke. The MAS was used to evaluate spasticity in ankle dorsiflexion, elbow flexion and knee extension (28). Participants lay supine position. A single trained physiotherapist moved the joint over ≈ 1 s and assigned a score (0, no increase in muscle tone; 1, slight increase in tone, manifested by a catch and release or minimal resistance at end-range; 1+, slight increase in tone, catch followed by minimal resistance through < 50% of range; 2, marked increase in tone through > 50% of range, but easy movement still possible; 3, considerable increase in tone, passive movement difficult; 4, affected part rigid in flexion or extension) (29, 30).

### 2.5 Statistical analyses

Statistical analyses were performed using SPSS Version 29 (IBM Corp., Armonk, NY, USA). All data were checked for compatibility with the assumptions of normality using the Shapiro–Wilk test. A chi-square (χ^2^) test was used to compare the proportions of sexes between the patient group and apparently healthy adults. For continuous variables, the independent t-test or Mann–Whitney U test was used to compare age, height, weight, BMI, Barthel Index, MAS score, MNA score, and MMSE score between the two groups. An analysis of covariance (ANCOVA) was conducted, with effect sizes interpreted using partial eta squared (ηp^2^). This analysis compared muscle quality and muscle strength across groups— participant after stroke (contralateral side vs. affected side) and apparently healthy adults—while controlling for potential confounders, including age, sex, nutritional status (BMI and MNA score), and cognitive performance (MMSE score). Cohen’s d coefficients were also reported to give an indication of effect size, considered as small (*d* ≥ 0.2), medium (*d* ≥ 0.5) and large (*d* ≥ 0.8) effects. Pearson product–moment correlation coefficients were calculated to assess associations between muscle quality parameters—EI and MT—measured in four muscles: BB, RF, TA, and GS. Relationships between muscle quality parameters, age, BMI, motor function, and muscle strength values were also assessed using Pearson product–moment correlation coefficients. Linear regression analysis, including age and sex as additional covariates, was conducted to assess the predictive ability of muscle parameters for motor functions. A *p*-value of <0.05 was considered statistically significant. A 95% confidence interval excluding 0 was also regarded as statistically significant.

## 3 Results

Table 1 presents the demographic and clinical data of the participants. A total of 102 participants were included: 49 people after stroke (51% men, mean age 62 ± 14 years) and 53 apparently healthy adults (55% women, mean age 66 ± 9 years). No significant differences were observed for height (166 ± 0.1 cm vs. 163 ± 0.1 cm, *p* = 0.119), weight (73.8 ± 15.1 kg vs. 71.4 ± 14.3 kg, *p* = 0.640), or BMI (26.5 ± 4.34 kg/m^2^ vs. 26.7 ± 4.42 kg/m^2^, *p* = 0.598). Functional scores differed markedly between groups: Barthel Index median (IQR) was 53 (25–80) vs. 99 (98–100), *p* < 0.001; EQ-5D VAS score was 6 ± 2.1 vs. 7.2 ± 1.51, *p* = 0.002; and EQ-5D-5L index value median (IQR) was 0.09 (0.09–0.53) vs. 0.91 (0.8–1.0), *p* < 0.001. Cognitive and nutritional assessments also favored controls: MMSE median (IQR) 26 (24–29) vs. 29 (28–30), *p* < 0.001; MNA 23 (19–26) vs. 28 (26–29), *p* < 0.001. In the stroke group, the median (IQR) MAS scores were 1 (0–3) for elbow flexors, 0 (0–1.5) for knee flexors, and 3 (0–3) for ankle flexors. Paretic side distribution was 43% right and 57% left, with an etiology of infarction in 51% and hemorrhage in 49%. Median post-stroke duration was 115 (36–143) months.

**Table 1:** Participant characteristics for people after stroke and apparently healthy adults.

Variable	People after stroke (*n* = 49)	Apparently healthy adults (*n* = 53)	*p* value	Cohen’s *d*
Sex (men/women), %	51/49	45/55	0.757^a^	−
Age, years	62 (14)	66 (9)	0.183^b^	0.266
Height, cm	166 (0.1)	163 (0.1)	0.119^b^	0.095
Weight, kg	73.8 (15.1)	71.4 (14.3)	0.640^b^	0.115
BMI, kg/m^2^	26.5 (4.34)	26.7 (4.42)	0.598^b^	0.107
Barthel index, score	53 (25–80)	99 (98–100)	<0.001^c^	0.451
EQ-5 (VAS), score	6 (2.1)	7.2 (1.51)	0.002^b^	0.655
EQ-5D-5L index value	0.09 (0.09–0.53)	0.91 (0.8–1.0)	<0.001^c^	0.549
MMSE, score	26 (24–29)	29 (28–30)	<0.001^c^	0.155
MNA, score	23 (19–26)	28 (26–29)	<0.001^c^	0.306
MAS elbow flexors, score	1 (0–3)	−	−	−
MAS knee flexors, score	0 (0–1.5)	−	−	−
MAS ankle flexors, score	3 (0–3)	−	−	−
Paretic side (right/left), %	43/57	−	−	−
Etiology (infarction/hemorrhage), %	51/49	−	−	−
Post stroke duration, months	115 (36–143)	−	−	−

Values are mean (SD) or median (percentile 25–75). BMI: Body mass index; EuroQol-5D (EQ-5D); VAS: Visual analog scale; MMSE: Mini Mental Status Evaluation; Modified Ashworth Scale (MAS); MNA: Mini Nutritional Assessment. Group differences were assessed using chi-square (χ^2^) testᵃ, independent t-test^b^ or the Mann–Whitney U test^c^.

**Figure 1:**
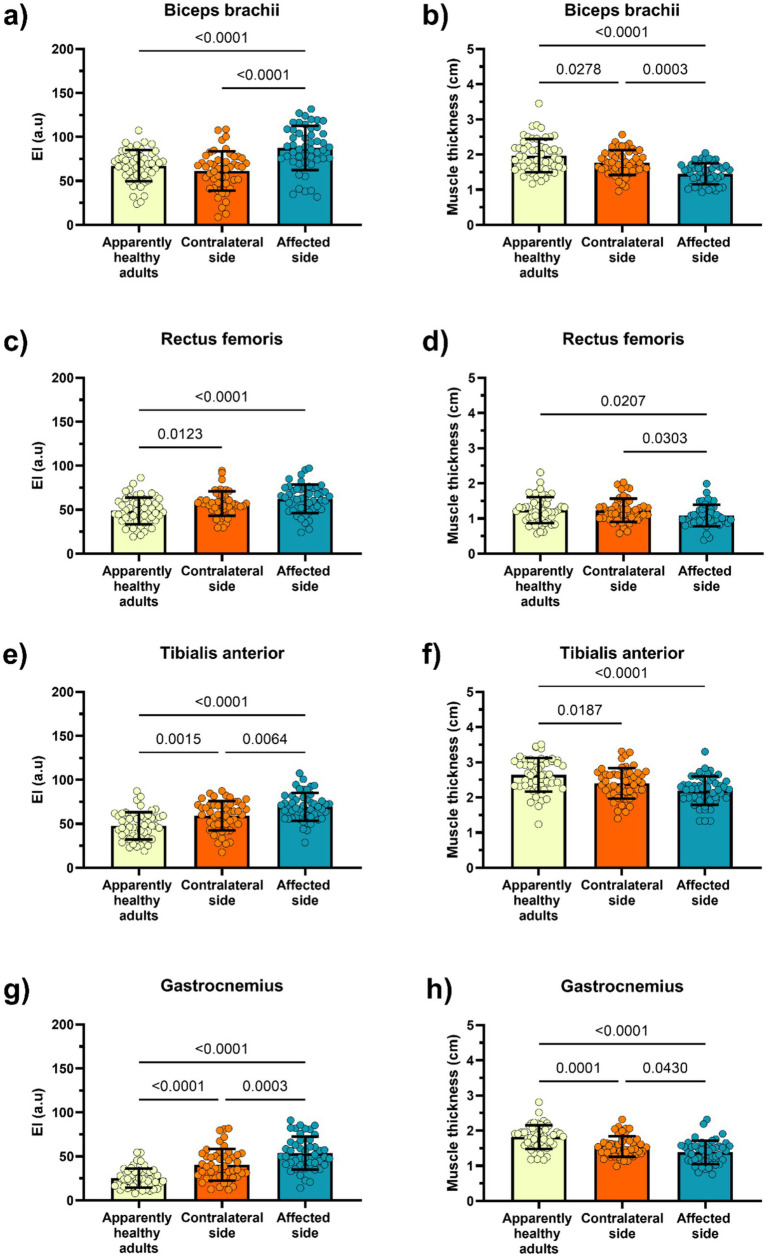
Echo intensity (arbitrary units) and muscle thickness (cm) values on the apparently healthy subjects and people after stroke (contralateral side vs. affected side). Raw data results are presented as mean Panels show group differences in echo intensity (EI, arbitrary units; **a, c, e, g)** and muscle thickness (cm; **b, d, f, h**) for the biceps brachii **(a, b)**, rectus femoris **(c, d)**, tibialis anterior **(e, f)**, and gastrocnemius **(g, h)**. Circles represent individual data points. P-values correspond to pairwise comparisons. Higher EI values indicate poorer muscle quality (greater intramuscular fat and connective tissue infiltration), whereas lower muscle thickness indicates reduced muscle mass. Fully adjusted means, SD, and *post hoc* contrasts from ANCOVA models are reported in Supplementary Table S2.

The raw data results are presented in Figure 1, with full estimates provided in Supplementary Table S2. Compared with apparently healthy individuals, participants post-stroke exhibited markedly higher EI in BB (+41.0%), RF (+32.9%), GS (+118.8%; ≈2.2-fold; *Δ* = +28.2 a.u.), and TA (+54.2%), accompanied by lower MT (−29.5, −12.9, −24.3, and −18.2%, respectively). Within the stroke group, the affected side demonstrated higher EI (+51.9%, +16.0%, +22.1%, +16.8%) and lower MT (−19.9%, −10.0%, −11.0%, −7.9%) across the same muscles relative to the contralateral side (Panels a–h; *p* < 0.05). In ANCOVA models adjusted for age, sex, BMI, nutritional status (MNA-SF), cognitive performance (MMSE), and time since stroke, the affected side consistently exhibited higher. People after stroke exhibited significantly higher EI and lower MT compared to apparently healthy adults across all assessed muscles (BB, RF, GS, TA), except for RF-MT values (*p* = 0.098). The affected side showed the most pronounced changes, with BB EI was +41% and TA EI + 54% higher than controls (*p* < 0.001), while MT reductions were most pronounced for BB (−29.5%) and GS (−24.3%). Within-stroke comparisons indicated that EI was significantly higher on the affected versus contralateral side for all muscles (*p* < 0.001), whereas MT differences were significant for BB and GS but not for TA after adjustment. ANCOVA models adjusting for age, sex, BMI, nutritional status, and cognition confirmed these differences, with the largest effect sizes for BB EI (*d* = 1.52) and BB MT (*d* = 1.50).

Supplementary Figures S1–S3 presents Spearman correlation coefficients (*r*_s_) for apparently healthy (Supplementary Figure S1) and people after stroke —contralateral side (Supplementary Figure S2) and affected side (Supplementary Figure S1)—based on age, sex, BMI, MNA-SF score, MMSE score, muscle quality, and muscle strength variables. The *r*-values revealed differing trends between apparently healthy and people after stroke on both sides. For example, in the apparently healthy group (Supplementary Figure S1), a significant moderate negative correlation was observed between the EI values of Limbs-1 and Limbs-2 and the MNA-SF score, MMSE score, handgrip strength, knee flexion strength, and MT values of the RF and TA (*r*_s_ range = −0.307 to −0.490, *p* < 0.05). By contrast, a moderate positive correlation was found between EI values and age: Limbs-1 (*r*_s_ = 0.524, *p* < 0.001) and Limbs-2 (*r*_s_ = 0.607, *p* < 0.001). Among people after stroke, although there were slight differences in correlation magnitude between the less affected and affected sides, the direction of the correlation coefficients remained consistent across all parameters (Supplementary Figures S2, S3). Muscle strength (grip strength, knee extension, knee flexion) showed strong positive correlations with MT of the BB (*r*_s_ range = 0.439–0.573, *p* < 0.001) and MT of the RF (*r*_s_ range = 0.325–0.439, *p* < 0.001). By contrast, EI of the RF showed moderate inverse correlations with grip strength (*r*_s_ = −0.385) on the contralateral side (Supplementary Figure S2). On the affected side, higher EI values in muscles such as the GS and RF were negatively correlated with the MNA-SF score (*r*_s_ range = −0.463 to −0.592), and muscle strength (e.g., EI of the RF with knee extension: *r*_s_ = −0.221) (Supplementary Figure S3).

**Figure 2:**
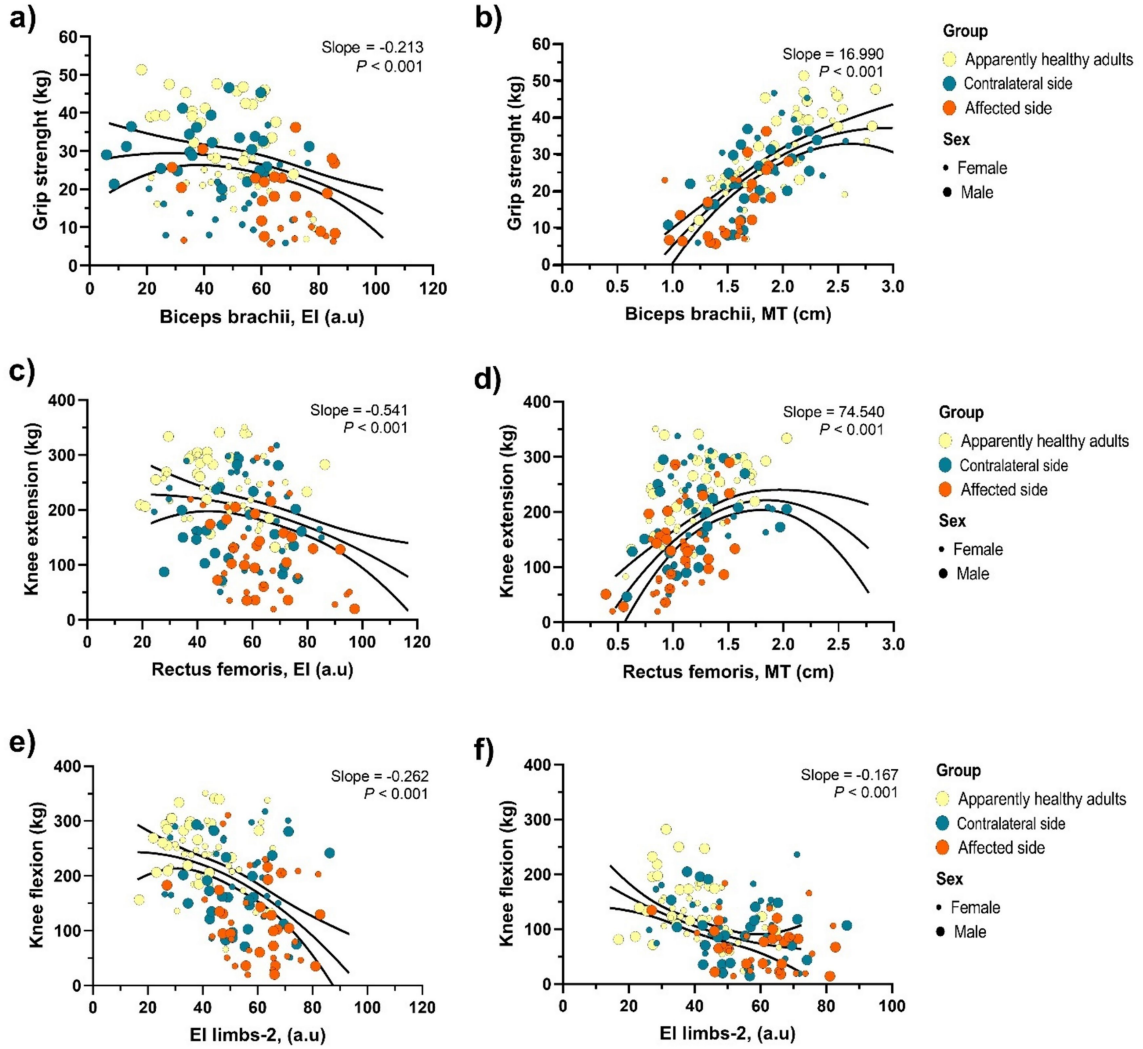
Relationships between muscle echo intensity, muscle thickness, and clinical variables in study participants. Panel **(a, c, e)**: Echo intensity (EI) in the biceps brachii, rectus femoris, and composite index (EI limbs-2) was negatively associated with grip strength, knee extension, and knee flexion strength (all *P* < 0.001). Panel **(b, d)**: Muscle thickness (MT) in the biceps brachii and rectus femoris was positively associated with grip strength and knee extension strength (all *P* < 0.001). Panel **(f)**: The composite index EI limbs-2 was inversely related to knee flexion strength (*P* < 0.001). Data points are color-coded by group: apparently healthy adults (yellow), contralateral side (green), and affected side (orange) and sex (female: smaller circles; male: larger circles). Black lines represent fitted regression slopes with 95% confidence intervals.

Figures 2a–f illustrates scatter plot associations between EI values and muscle strength variables across all participant groups. EI values of the BB were negatively correlated with handgrip strength (slope = −0.213, *p* < 0.001) (Figure 2a), while MT values of the BB were positively associated with handgrip strength (slope = 16.990, *p* < 0.001) (Figure 2b), suggesting a relationship between muscle quality and upper limb function. In Figure 2c, a significant negative association was observed between EI values of the RF and knee extension strength (slope = −0.541, *p* < 0.001). Conversely, Figure 2d shows a significant positive association between RF MT and knee extension strength (slope = 74.540, *p* < 0.001), with greater MT consistently linked to better lower-limb strength outcomes. In Figure 2e, EI values of Limbs-2 exhibited a moderate negative association with knee flexion strength (slope = −0.262, *p* < 0.001), indicating that increased muscle EI—commonly associated with reduced muscle quality—corresponds with diminished lower-limb strength. Figure 2f confirms this trend, showing a slightly weaker but still significant correlation (slope = −0.167, *p* < 0.001). These findings further support the inverse association between muscle quality, as reflected by higher EI values, and functional motor performance, particularly when evaluated using limb muscle quality across both apparently healthy subjects and people after stroke.

## 4 Discussion

There is a critical need for practical methods to monitor muscle quality changes after stroke. Ultrasound emerges as a suitable option given its portability and clinical feasibility. In this study, people after stroke displayed markedly higher EI in BB, RF, TA, and GS, accompanied by reduced MT, particularly on the affected side. EI was inversely associated with handgrip and knee extension strength, while MT showed strong positive correlations with strength outcomes. These findings underscore regional muscle quality deterioration and its direct impact on functional capacity.

In terms of morphometric changes, significant reductions in MT were observed in the BB, TA, and GS muscles on the affected side, consistent with disuse atrophy and impaired neuromuscular activation following stroke (31–33). Our results are consistent with earlier studies reporting increased intramuscular fat and fibrosis in paretic muscles, contributing to impaired contractility and metabolic inefficiency. For example, Nozoe et al. (34) reported decreased MT and increased EI in both quadriceps (RF) of people after stroke. Similarly, Monjo et al. (20) found that in lower-leg muscles on the paretic side, MT values were significantly lower, while EI values of the TA and GS were significantly higher in people after stroke than in healthy adults. In part, our hypothesis was confirmed because people after stroke exhibited reduced MT and increased EI compared with apparently healthy. This is likely due to muscle atrophy in the limbs, which commonly occurs as a result of impairments in the central nervous system (16). The stiffness of spastic muscle at rest depends on both muscle reflex mechanisms—where hyperexcitable reflexes may be triggered by even small movements—and changes in the passive mechanical properties of the muscle (5, 35). As stiffness progressively increases, this loss of range of motion further contributes to impaired limb and motor function (36). Basic pathological studies have shown a specific loss of slow-twitch fibres on the paretic side. For example, Hafer-Macko et al. (37) found that muscles in the lower limbs tend to have a higher proportion of slow-twitch fibres, whereas thigh muscles are predominantly composed of fast-twitch fibres. This muscle fibre composition may help explain the observed reductions in MT and elevations in EI in the limb muscles of people after stroke. Mechanistically, these alterations likely reflect the combined effects of disuse, diminished central drive, spasticity-related architectural changes, and fat–fibrosis infiltration within skeletal muscle. These alterations likely indicate preferential atrophy of contractile fibres, accompanied by a relative accumulation of non-contractile elements—such as adipose tissue and fibrous components—within the muscle structure following a stroke (32, 38–42). The observed increase in EI likely reflects IMAT and fibrotic infiltration, while reduced MT indicates structural atrophy. These changes are driven by disuse, spasticity-induced architectural alterations, and neuromuscular denervation, which collectively perpetuate a cycle of functional decline. This phenomenon aligns with the broader concept of the “metabaging cycle,” wherein systemic inflammation, insulin resistance, and mitochondrial dysfunction accelerate sarcopenic processes (42). Rehabilitation strategies should therefore integrate progressive resistance training, neuromuscular stimulation, and nutritional optimization to mitigate atrophy and promote muscle remodeling. High-velocity resistance exercise and functional electrical stimulation have shown promise in enhancing muscle architecture and motor recovery (43, 44).

The correlational analyses reinforce the clinical relevance of muscle quality measures. In apparently healthy adults, EI values from global limb indices (Limbs-1 and Limbs-2) were moderately and negatively correlated with the nutritional status (MNA-SF score), cognitive performance (MMSE score), and functional strength measures (handgrip, knee flexion, and MT values of RF and TA). Moreover, positive associations between EI values and age were found, consistent with previous literature describing age-related changes in muscle composition, including increased intramuscular fat and fibrosis. These findings align with prior research by Watanabe et al. (39) and Reimers et al. (40), who also documented age-associated increases in muscle EI alongside reductions in motor function. Conversely, in people after stroke, although slight differences in the strength of associations were observed between the affected and less affected limbs, the overall direction of the relationships remained consistent. On the contralateral side, increased EI values were moderately and inversely correlated with handgrip strength and Barthel Index scores. On the affected side, higher EI in the GS and RF muscles was negatively associated with the nutritional status (as measured by the MNA-SF score), and lower-limb strength. These findings indicate that EI correlates with muscle-quality deterioration and displays functional associations on both sides in people after stroke. The linear regression analysis further reinforced the clinical relevance of ultrasound-derived muscle measurements. In particular, elevated EI in the BB, RF, and Limbs-2 regions was consistently associated with lower grip and knee strength values, while increased MT was linked to better muscle strength. In our analysis, individual muscle EI measures demonstrated stronger effect sizes and higher correlations with clinical outcomes compared with the composite indices, indicating limited utility in combining multiple muscle sites such as EI limbs-1 and/or EI limbs-2. In this context, integrating EI values across different muscles and limb regions did not provide a more robust or reliable indicator of overall muscle quality deterioration. These findings suggest that, in our cohort, creating composite indices from multiple muscles did not enhance the robustness or predictive power of ultrasound-based muscle quality assessments in people after stroke.

Thus, in people after stroke, the relationship between quantitative and qualitative muscle changes may differ by muscle, independent of age, sex, BMI, MNA-SF score, and MMSE score. For example, spastic equinus can restrict gastrocnemius activation, accelerating fatty and fibrous tissue infiltration, whereas the quadriceps often retains partial activation during daily transfers, mitigating the extent of deterioration (41). These findings highlight the complementary roles of muscle mass and quality in determining functional motor performance and overall health status in both apparently healthy individuals and those post-stroke. Importantly, the observed associations between ultrasound-derived parameters (EI and MT) and strength outcomes reinforce the clinical relevance of muscle quality as a surrogate marker for motor function, suggesting that these measures could aid in stratifying rehabilitation priorities and tailoring intervention strategies.

There are certain limitations to acknowledge in this study. First, the sample consisted solely of individuals aged ≥40 years who were receiving rehabilitation services through the ADACEN long-term care insurance system. This recruitment approach may limit the applicability of the results to other populations, and future studies should consider more diverse groups to improve external validity. Second, the relatively limited sample size may have reduced the ability to detect some associations, leaving room for potential type II errors. For example, the effects of age, sex, and clinical status on the differences between people after stroke and apparently healthy may not have been fully accounted for. Third, the MT and EI values in the apparently healthy group do not necessarily represent normative values across the age ranges. Fourth, prior research has demonstrated that physical activity levels are strongly associated with MT and EI in both apparently healthy adults (45, 46) and people after stroke (47). Fifth, although muscle architecture includes parameters such as fascicle length and pennation angle, this study focused on ultrasound-derived measures of MT and EI. This represents a limitation, particularly given the relevance of muscle shortening, which is a key feature of chronic post-stroke muscle change (48–51). Additional research is warranted to better understand its potential impact. Finally, future studies should investigate the relationship between muscle quality parameters and stroke-related neuromuscular manifestations, such as diminished contractile function and spasticity.

Collectively, our results highlight the practical relevance of incorporating muscle ultrasound into the clinical evaluation of individuals with neurological conditions. Muscle morphological parameters—particularly MT and EI—appear to be robust, non-invasive markers of muscle architecture, showing meaningful associations with both functional capacity and health related quality of life. In conclusion, this study demonstrates that people after stroke experience localized alterations in both muscle mass and muscle quality. The findings offer valuable insights for the evaluation and design of targeted interventions, such as physical training or dietary support, aimed at enhancing muscle function in this population.

## Data Availability

The original contributions presented in the study are included in the article/Supplementary material, further inquiries can be directed to the corresponding author.

## References

[1] Feigin VL Abate MD Abate YH Abd ElHafeez S Abd-Allah F Abdelalim A. Global, regional, and national burden of stroke and its risk factors, 1990–2021: a systematic analysis for the global burden of disease study 2021. Lancet Neurol. (2024) 23:973–1003. doi: 10.1016/S1474-4422(24)00369-7, PMID: 39304265 PMC12254192

[2] Gil-Salcedo A Dugravot A Fayosse A Landré B Jacob L Bloomberg M. Pre-stroke disability and long-term functional limitations in stroke survivors: findings from more of 12 years of follow-up across three international surveys of aging. Front Neurol. (2022) 13:888119. doi: 10.3389/fneur.2022.888119, PMID: 35775052 PMC9237334

[3] Harris ML Polkey MI Bath PM Moxham J. Quadriceps muscle weakness following acute hemiplegic stroke. Clin Rehabil. (2001) 15:274–81. doi: 10.1191/026921501669958740, PMID: 11386397

[4] Fukumoto Y Ikezoe T Yamada Y Tsukagoshi R Nakamura M Takagi Y. Age-related ultrasound changes in muscle quantity and quality in women. Ultrasound Med Biol. (2015) 41:3013–7. doi: 10.1016/j.ultrasmedbio.2015.06.017, PMID: 26278633

[5] English CK Thoirs KA Fisher L McLennan H Bernhardt J. Ultrasound is a reliable measure of muscle thickness in acute stroke patients, for some, but not all anatomical sites: a study of the intra-rater reliability of muscle thickness measures in acute stroke patients. Ultrasound Med Biol. (2012) 38:368–76. doi: 10.1016/j.ultrasmedbio.2011.12.012, PMID: 22266233

[6] Tomeleri CM Cavalcante EF Antunes M Nabuco HC de Souza MF Teixeira DC. Phase angle is moderately associated with muscle quality and functional capacity, independent of age and body composition in older women. J Geriatr Phys Ther. (2019) 42:281–6. doi: 10.1519/JPT.0000000000000161 29210931

[7] Chon J Soh Y Shim GY. Stroke-related sarcopenia: pathophysiology and diagnostic tools. Brain Neurorehabilitation. (2024) 17:e23. doi: 10.12786/bn.2024.17.e23, PMID: 39649713 PMC11621676

[8] Akazawa N Harada K Okawa N Tamura K Moriyama H. Muscle mass and intramuscular fat of the quadriceps are related to muscle strength in non-ambulatory chronic stroke survivors: A cross-sectional study. PLoS One. (2018) 13:e0201789. doi: 10.1371/journal.pone.0201789, PMID: 30071100 PMC6072321

[9] Pradines M Jabouille F Fontenas E Baba Aissa I Gault-Colas C Baude M. Does spastic myopathy determine active movement and ambulation speed in chronic spastic paresis?-A cross-sectional study on plantar flexors. PLoS One. (2024) 19:e0310969. doi: 10.1371/journal.pone.0310969, PMID: 39446866 PMC11500935

[10] Irisawa H Mizushima T. Assessment of changes in muscle mass, strength, and quality and activities of daily living in elderly stroke patients. Int J Rehabil Res. (2022) 45:161–7. doi: 10.1097/MRR.0000000000000523, PMID: 35170496 PMC9071026

[11] Pang MY Eng JJ McKay HA Dawson AS. Reduced hip bone mineral density is related to physical fitness and leg lean mass in ambulatory individuals with chronic stroke. Osteoporos Int. (2005) 16:1769–79. doi: 10.1007/s00198-005-1925-1, PMID: 15902416 PMC3145668

[12] Suzuki K Nakamura R Yamada Y Handa T. Determinants of maximum walking speed in hemiparetic stroke patients. Tohoku J Exp Med. (1990) 162:337–44. doi: 10.1620/tjem.162.337, PMID: 2102565

[13] Prado-Medeiros CL Silva MP Lessi GC Alves MZ Tannus A Lindquist AR. Muscle atrophy and functional deficits of knee extensors and flexors in people with chronic stroke. Phys Ther. (2012) 92:429–39. doi: 10.2522/ptj.20090127, PMID: 22135704

[14] Ishimoto T Taniguchi Y Akazawa N. Longitudinal relationship between intramuscular fat in the quadriceps and gait Independence in convalescent stroke patients. J Stroke Cerebrovasc Dis. (2020) 29:105287. doi: 10.1016/j.jstrokecerebrovasdis.2020.105287, PMID: 33066923

[15] Thielman G Yourey L. Ultrasound imaging of upper extremity spastic muscle post-stroke and the correlation with function: A pilot study. NeuroRehabilitation. (2019) 45:213–220. doi: 10.3233/NRE-192742, PMID: 31498134

[16] Nelson CM Murray WM Dewald JPA. Motor impairment-related alterations in biceps and triceps Brachii fascicle lengths in chronic Hemiparetic stroke. Neurorehabil Neural Repair. (2018) 32:799–809. doi: 10.1177/1545968318792618, PMID: 30136897 PMC6296776

[17] Ryan AS Buscemi A Forrester L Hafer-Macko CE Ivey FM. Atrophy and intramuscular fat in specific muscles of the thigh: associated weakness and hyperinsulinemia in stroke survivors. Neurorehabil Neural Repair. (2011) 25:865–72. doi: 10.1177/1545968311408920, PMID: 21734070 PMC3546168

[18] Harris-Love MO Avila NA Adams B Zhou J Seamon B Ismail C. The comparative associations of ultrasound and computed tomography estimates of muscle quality with physical performance and metabolic parameters in older men. J Clin Med. (2018) 7:E340. doi: 10.3390/jcm7100340, PMID: 30308959 PMC6210142

[19] Reimers K Reimers CD Wagner S Paetzke I Pongratz DE. Skeletal muscle sonography: a correlative study of echogenicity and morphology. J Ultrasound Med. (1993) 12:73–777. doi: 10.7863/jum.1993.12.2.73 8468739

[20] Monjo H Fukumoto Y Asai T Kubo H Ohshima K Tajitsu H. Differences in muscle thickness and echo intensity between stroke survivors and age- and sex-matched healthy older adults. Phys Ther Res. (2020) 23:188–94. doi: 10.1298/ptr.E10018, PMID: 33489658 PMC7814197

[21] Hunnicutt JL Gregory CM. Skeletal muscle changes following stroke: a systematic review and comparison to healthy individuals. Top Stroke Rehabil. (2017) 24:463–71. doi: 10.1080/10749357.2017.1292720, PMID: 28251861 PMC5801663

[22] Young HJ Jenkins NT Zhao Q Mccully KK. Measurement of intramuscular fat by muscle echo intensity. Muscle Nerve. (2015) 52:963–71. doi: 10.1002/mus.24656, PMID: 25787260 PMC4575231

[23] Caresio C Molinari F Emanuel G Minetto MA. Muscle echo intensity: reliability and conditioning factors. Clin Physiol Funct Imaging. (2015) 35:393–403. doi: 10.1111/cpf.12175, PMID: 24902991

[24] Yoshiko A Kaji T Sugiyama H Koike T Oshida Y Akima H. Muscle quality characteristics of muscles in the thigh, upper arm and lower back in elderly men and women. Eur J Appl Physiol. (2018) 118:1385–1395. doi: 10.1007/s00421-018-3870-7, PMID: 29687267

[25] MacDermid J Solomon G Fedorczyk J Valdes K. Clinical assessment recommendations American Society of Hand Therapists (2015).

[26] Quinn TJ Langhorne P Stott DJ. Barthel index for stroke trials: development, properties, and application. Stroke. (2011) 42:1146–51. doi: 10.1161/STROKEAHA.110.598540, PMID: 21372310

[27] Cioncoloni D Piu P Tassi R Acampa M Guideri F Taddei S. Relationship between the modified Rankin scale and the Barthel index in the process of functional recovery after stroke. NeuroRehabilitation. (2012) 30:315–22. doi: 10.3233/NRE-2012-0761, PMID: 22672946

[28] Meseguer-Henarejos AB Sánchez-Meca J López-Pina JA Carles-Hernández R. Inter- and intra-rater reliability of the modified Ashworth scale: a systematic review and meta-analysis. Eur J Phys Rehabil Med. (2018) 54:576–90. doi: 10.23736/S1973-9087.17.04796-7, PMID: 28901119

[29] Bohannon RW Smith MB. Interrater reliability of a modified Ashworth scale of muscle spasticity. Phys Ther. (1987) 67:206–7. doi: 10.1093/ptj/67.2.206, PMID: 3809245

[30] Ansari NN Naghdi S Arab TK Jalaie S. The interrater and intrarater reliability of the modified Ashworth scale in the assessment of muscle spasticity: limb and muscle group effect. NeuroRehabilitation. (2008) 23:231–7. doi: 10.3233/NRE-2008-23304, PMID: 18560139

[31] Yang J Jiang F Yang M Chen Z. Sarcopenia and nervous system disorders. J Neurol. (2022) 269:5787–97. doi: 10.1007/s00415-022-11268-8, PMID: 35829759

[32] Scherbakov N Von Haehling S Anker SD Dirnagl U Doehner W. Stroke induced sarcopenia: muscle wasting and disability after stroke. Int J Cardiol. (2013) 170:89–94. doi: 10.1016/j.ijcard.2013.10.031, PMID: 24231058

[33] Parry SM Puthucheary ZA. The impact of extended bed rest on the musculoskeletal system in the critical care environment. Extrem Physiol Med. (2015) 4:16. doi: 10.1186/s13728-015-0036-7, PMID: 26457181 PMC4600281

[34] Nozoe M Kanai M Kubo H Kitamura Y Yamamoto M Furuichi A. Changes in quadriceps muscle thickness, disease severity, nutritional status, and C-reactive protein after acute stroke. J Stroke Cerebrovasc Dis. (2016) 25:2470–4. doi: 10.1016/j.jstrokecerebrovasdis.2016.06.020, PMID: 27388709

[35] Dietz V Sinkjaer T. Spastic movement disorder: impaired reflex function and altered muscle mechanics. Lancet Neurol. (2007) 6:725–33. doi: 10.1016/S1474-4422(07)70193-X, PMID: 17638613

[36] Roots J Trajano GS Fontanarosa D. Ultrasound elastography in the assessment of post-stroke muscle stiffness: a systematic review. Insights Imaging. (2022) 13:67. doi: 10.1186/s13244-022-01191-x, PMID: 35380302 PMC8982789

[37] Hafer-Macko CE. Skeletal muscle changes after hemiparetic stroke and potential beneficial effects of exercise intervention strategies. J Rehabil Res Dev. (2008) 45:261–72. doi: 10.1682/JRRD.2007.02.0040, PMID: 18566944 PMC2978978

[38] English C McLennan H Thoirs K Coates A Bernhardt J. Loss of skeletal muscle mass after stroke: A systematic review. Int J Stroke. (2010) 5:395–402. doi: 10.1111/j.1747-4949.2010.00467.x, PMID: 20854624

[39] Watanabe Y Ikenaga M Yoshimura E Yamada Y Kimura M. Association between echo intensity and attenuation of skeletal muscle in young and older adults: a comparison between ultrasonography and computed tomography. Clin Interv Aging. (2018) 13:1871–8. doi: 10.2147/CIA.S173372, PMID: 30323573 PMC6174294

[40] Reimers CD Harder T Saxe H. Age-related muscle atrophy does not affect all muscles and can partly be compensated by physical activity: an ultrasound study. J Neurol Sci. (1998) 159:60–6. doi: 10.1016/S0022-510X(98)00134-8, PMID: 9700705

[41] Beyaert C Vasa R Frykberg GE. Gait post-stroke: pathophysiology and rehabilitation strategies. Neurophysiol Clin. (2015) 45:335–55. doi: 10.1016/j.neucli.2015.09.005 26547547

[42] Cheng Y Liu R Wang RR Yu K Shen J Pang J. The metabaging cycle promotes non-metabolic chronic diseases of ageing. Cell Prolif. (2024) 57:e13712. doi: 10.1111/cpr.13712, PMID: 38988247 PMC11471437

[43] Fukumoto Y Yamada Y Ikezoe T Watanabe Y Taniguchi M Sawano S. Association of physical activity with age-related changes in muscle echo intensity in older adults: a 4-year longitudinal study. J Appl Physiol. (2018) 125:1468–74. doi: 10.1152/japplphysiol.00317.2018, PMID: 30113271

[44] Izquierdo M de Souto Barreto P Arai H Bischoff-Ferrari HA Cadore EL Cesari M. Global consensus on optimal exercise recommendations for enhancing healthy longevity in older adults (ICFSR). J Nutr Health Aging. (2025) 29:100401. doi: 10.1016/j.jnha.2024.100401, PMID: 39743381 PMC11812118

[45] Saunders DH Sanderson M Hayes S Johnson L Kramer S Carter DD. Physical fitness training for stroke patients. Cochrane Database Syst Rev. (2020) 3:CD003316. doi: 10.1002/14651858.CD003316.pub7, PMID: 32196635 PMC7083515

[46] Farr JN Van Loan MD Lohman TG Going SB. Lower physical activity is associated with skeletal muscle fat content in girls. Med Sci Sports Exerc. (2012) 44:1375–81. doi: 10.1249/MSS.0b013e31824749b2, PMID: 22217562 PMC3819115

[47] English C Healy GN Coates A Lewis L Olds T Bernhardt J. Sitting and activity time in people with stroke. Phys Ther. (2016) 96:193–201. doi: 10.2522/ptj.20140522, PMID: 26112254

[48] Gao F Grant TH Roth EJ Zhang LQ. Changes in passive mechanical properties of the gastrocnemius muscle at the muscle fascicle and joint levels in stroke survivors. Arch Phys Med Rehabil. (2009) 90:819–26. doi: 10.1016/j.apmr.2008.11.004, PMID: 19406302

[49] Wang R Zhang L Jalo H Tarassova O Pennati GV Arndt A. Individualized muscle architecture and contractile properties of ankle plantarflexors and dorsiflexors in post-stroke individuals. Front Bioeng Biotechnol. (2024) 12:1453604. doi: 10.3389/fbioe.2024.1453604, PMID: 39659988 PMC11628271

[50] Zhao H Ren Y Roth EJ Harvey RL Zhang LQ. Concurrent deficits of soleus and gastrocnemius muscle fascicles and Achilles tendon post stroke. J Appl Physiol (1985). (2015) 118:863–71. doi: 10.1152/japplphysiol.00226.2014, PMID: 25663670 PMC4385882

[51] Pradines M Ghédira M Bignami B Vielotte J Bayle N Marciniak C. Do muscle changes contribute to the neurological disorder in spastic paresis? Front Neurol. (2022) 13:817229. doi: 10.3389/fneur.2022.817229, PMID: 35370894 PMC8964436

