# Supplementary material for: Differences in muscle quality, muscle strength, and functional motor performance between people after stroke and age- and sex-matched apparently healthy adults: a cross-sectional study
Source: Front Neurol. 2025 Sep 9;16:1641859. doi: 10.3389/fneur.2025.1641859 (PMC12454060; doi:10.3389/fneur.2025.1641859)
Supplement: Supplementary file 1 [file Data_Sheet_1.PDF]

Supplementary Table S1. Ultrasound measurement sites and standardized participant positions for assessed muscles.

**Muscles**	**Position**	**Measurement sites**
Biceps brachii (BB)	Supine	Perpendicular to the muscle mass, 5 cm from the elbow crease on the lateral column of the biceps to capture the long head of the biceps (1,2).
Tibialis anterior (TA)	Supine	The ultrasound head is placed 5 cm distally from the head of the fibula. Choosing the measurement area m1 of Martin-Rodriguez et al. (3) and Molinari et al. (4)
Rectus femoris (RF)	Supine	At the midpoint between the anterior superior iliac spine and the superior pole of the patella (5,6). It is also defined as the best assessment point in stroke patients(7).
Medial gastrocnemius (GS)	Sitting	Medial head of gastrocnemius at 30% proximal between the lateral malleolus of the fibula and the lateral condyle of the femur (3,5).

References

1. Asakawa DS, Pappas GP, Drace JE, Delp SL. Aponeurosis length and fascicle insertion angles of the biceps brachii. J Mech Med Biol. 2002;02(03n04):449-55.

2. Nelson CM, Dewald JPA, Murray WM. In vivo measurements of biceps brachii and triceps brachii fascicle lengths using extended field-of-view ultrasound. J Biomech. 2016;49(9):1948-52.

3. Martin-Rodriguez S, Gonzalez-Henriquez JJ, Diaz-Conde JC, Calbet JAL, Sanchis-Moysi J. The relationship between muscle thickness and pennation angle is mediated by fascicle length in the muscles of the lower extremities. Sci Rep. 2024;14(1).

4. Molinari F, Caresio C, Acharya UR, Mookiah MRK, Minetto MA. Advances in Quantitative Muscle Ultrasonography Using Texture Analysis of Ultrasound Images. Ultrasound Med Biol. 2015;41(9):2520-32.

5. Hafızoğlu M, Yıldırım HK, Okyar Baş A, Karaduman D, Şahiner Z, Doğu BB, et al. Role of muscle ultrasound in frailty assessment in older adults with type 2 diabetes mellitus. BMC Geriatr. 2024;24(1).

6. Mogi Y, Wakahara T. Effects of growth on muscle architecture of knee extensors. J Anat. 2022;241(3):683-91.

7. Monte A, Franchi MV. Regional muscle features and their association with knee extensors force production at a single joint angle. Eur J Appl Physiol. 2023;123(10):2239-48.

Supplementary Table S2. Full adjusted means, standard errors, and *post hoc* contrasts

Muscle	Parameter	People after stroke (n=49)		Apparently healthy subject (n=53)	Between groups differences adjusted*			Effect size model	
		Mean (SD) adjusted*			P value (Cohen *d*)			η²	F
		Affected side	Contralateral side	Dominant side	Control vs Affected side	Control vs Contralateral side	Affected side vs Contralateral side		
BB	EI (AU)	71.061 (2.91)	46.55 (2.50)	46.50 (2.65)	< 0.001 (*d* =1.523)	1 (*d* = 0.003)	< 0.001 (*d* =1.520)	0.268	23.987
	MT (cm)	1.43 (0.07)	1.75 (0.05)	1.99 (0.061)	< 0.001 (*d* =1.501)	0.019 (*d* = 0.643)	0.001 (*d* = 0.858)	0.189	15.165
RF	EI (AU)	57.15 (2.37)	53.74 (2.02)	53.75 (2.2)	1 (*d* = 0.260)	1 (*d* =0.0004	0.792 (*d* = 0.260)	0.011	0.685
	MT (cm)	1.18 (0.05)	1.22 (0.04)	1.205 (0.053)	1 (*d* = 0.078)	1 (*d* = 0.066)	1 (*d* = 0.144)	0.003	0.192
GS	EI (AU)	45.58 (2.90)	40.16 (2.38)	28.95 (2.5)	0.001 (*d* =1.075)	0.007 (*d* = 0.724)	0.416 (*d* = 0.351)	0.113	7.966
	MT (cm)	1.49 (0.06)	1.53 (0.04)	1.76 (0.052)	0.008 (*d* = 0.848)	0.007 (*d* = 0.721)	1 (*d* =0.127)	0.133	9.850
TA	EI (AU)	65.82 (2.77)	59.43(2.37)	49.7 (2.55)	0.001 (*d* = 1.041)	0.022 (*d* = 0.628)	0.229 (*d* = 0.413)	0.101	7.293
	MT (cm)	2.22 (0.07)	2.37 (0.06)	2.69 (0.069)	< 0.001 (*d* = 1.138)	0.003 (*d* = 0.78)	0.379 (*d* = 0.358)	0.123	9.069
EI LIMB 1		58.47 (1.95)	50.66 (1.67)	43.85 (1.79)	< 0.001 (*d* = 1.346)	0.023 (*d* = 0.626)	0.007 (*d* = 0.720)	0.155	11.934
EI LIMB 2		56.54 (2.16)	51.89 (1.82)	42.33 (1.9)	< 0.001 (*d* = 1.222)	0.002 (*d* = 0.823)	0.273 (*d* = 0.399)	0.140	10.201

Legend: *An analysis of covariance (ANCOVA) was conducted, with effect sizes interpreted using partial eta squared (ηp²). This analysis compared muscle quality and muscle strength across groups— participant after stroke (contralateral side vs. affected side) and apparently healthy adults—while controlling for potential confounders, including age, sex, nutritional status (BMI and MNA score), and cognitive performance (MMSE score). Cohen's d effect sizes were also reported to give an indication of effect size, considered as small ($d \geq 0.2$), medium ($d \geq 0.5$) and large ($d \geq 0.8$) effects. The ηp^2 values were interpreted as follows: small (≥ 0.01), moderate (≥ 0.06), and large (≥ 0.14) main effects.

Supplementary Figures S1-S3. Heatmap correlation among clinical, muscle strength, muscle thickness (MT), and echo intensity (EI) from apparently healthy subjects (**Figure S1**) and people after stroke (contralateral side, **Figure S2**) and affected side (**Figure S3**).

Figure S1. Apparently healthy subjects.

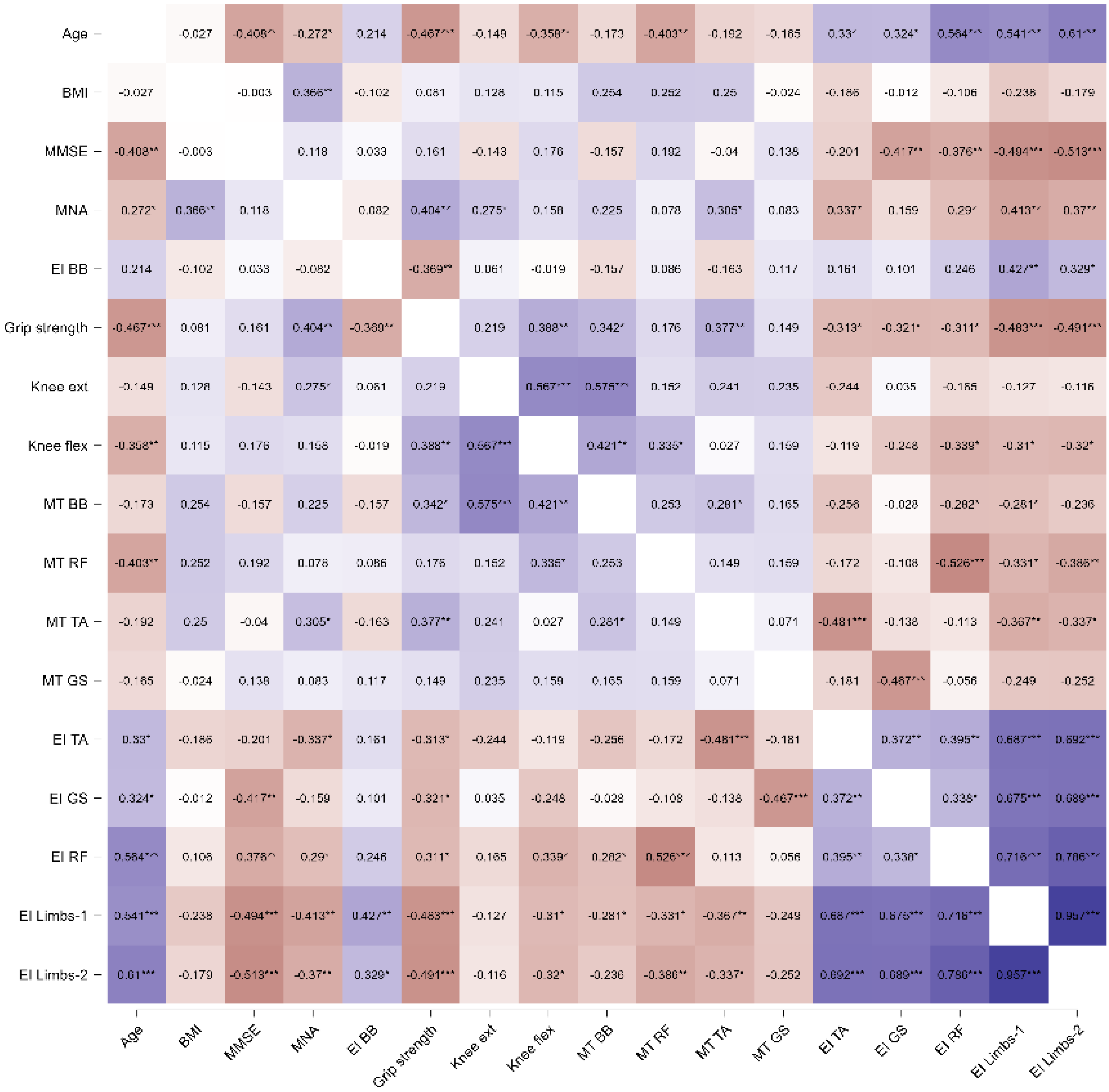

Legend: * p<0.05; ** p<0.01; *** p<0.001.

Figure S2. People after stroke (contralateral side)

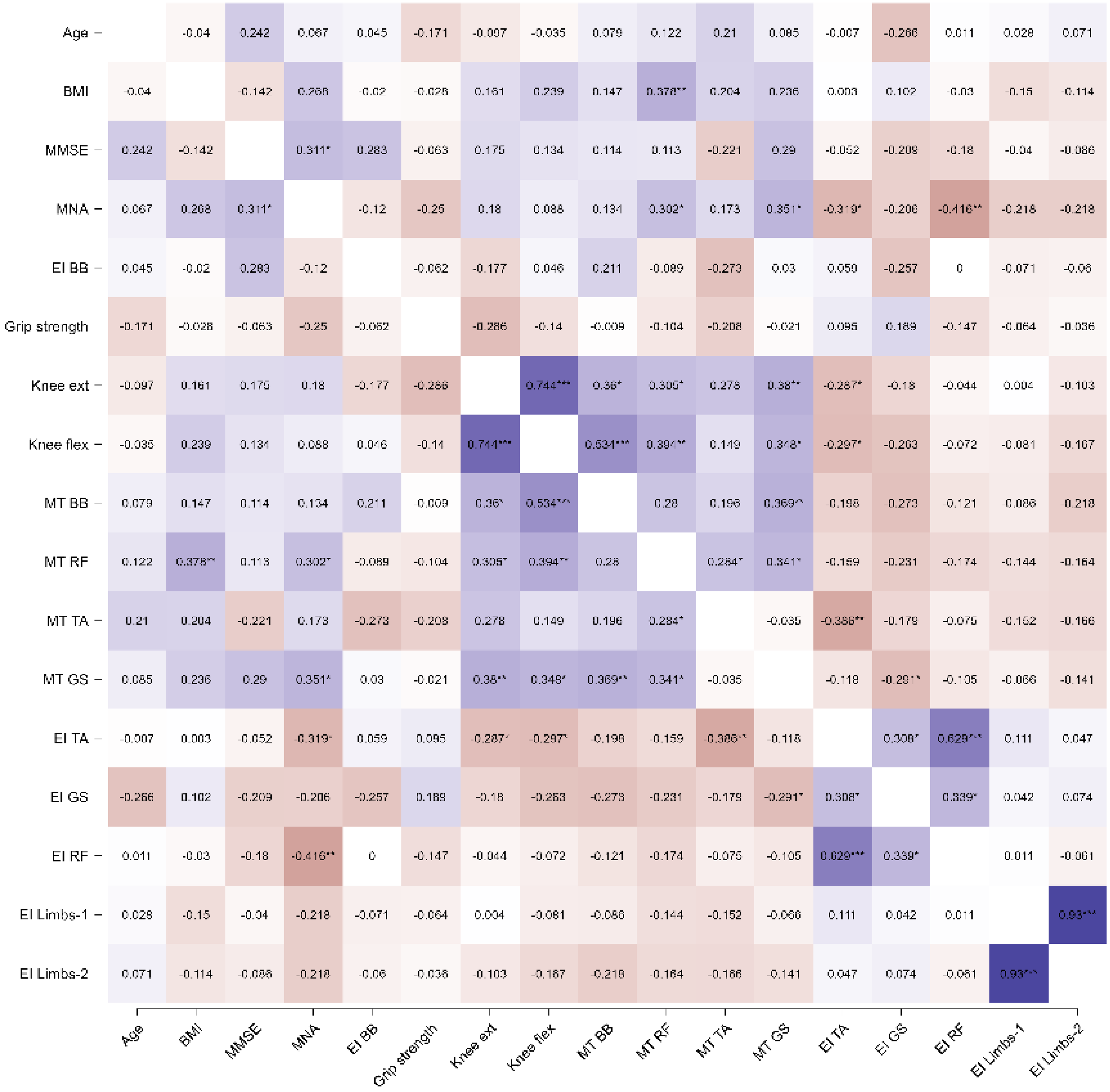

Legend: * p<0.05; ** p<0.01; *** p<0.001.

Figure S3. People after stroke (affected side)

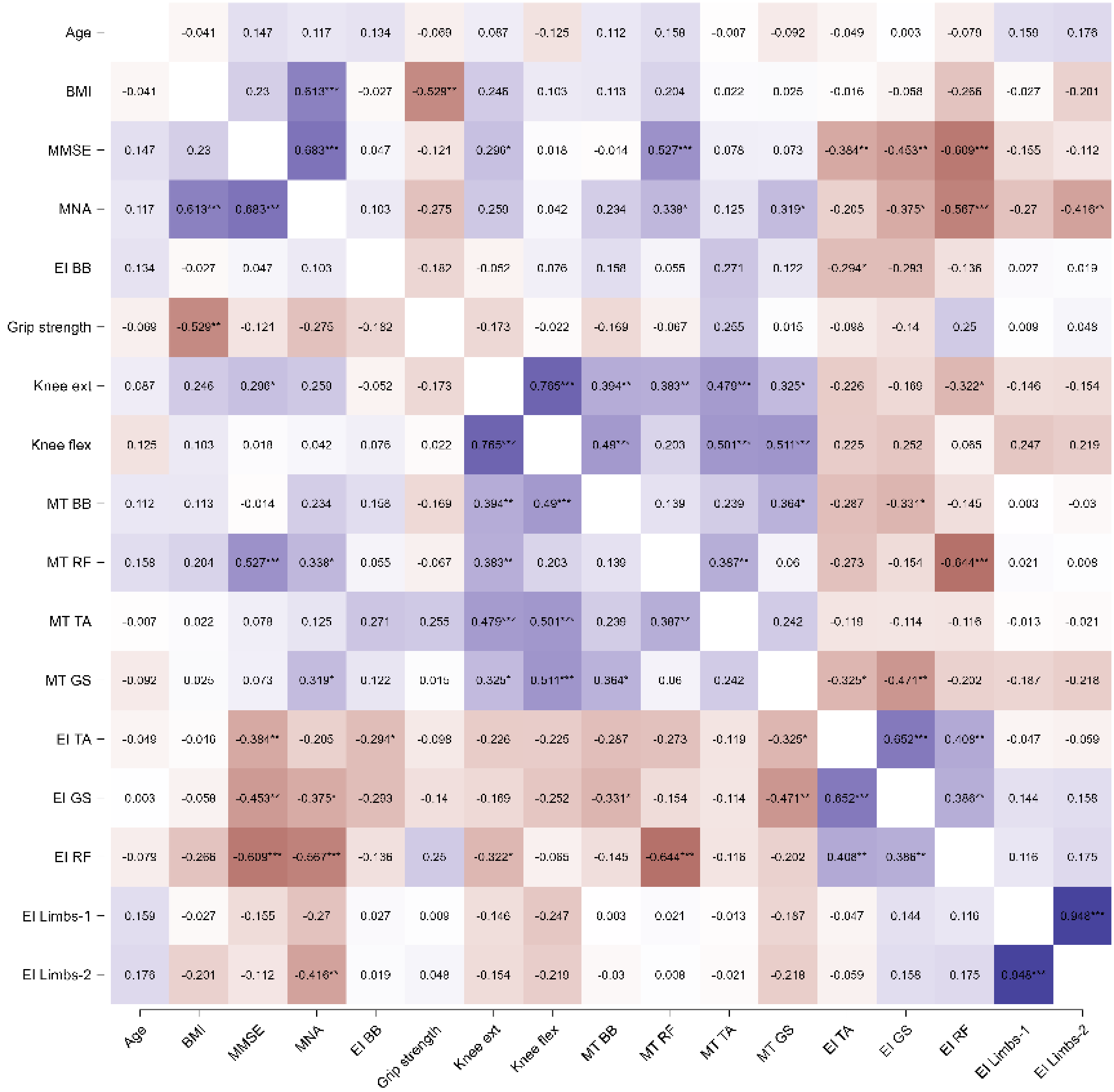

Legend: * p<0.05; ** p<0.01; *** p<0.001.
